# Effects of household washing on bacterial load and removal of *Escherichia coli* from lettuce and “ready‐to‐eat” salads

**DOI:** 10.1002/fsn3.514

**Published:** 2017-09-20

**Authors:** Elisabeth Uhlig, Crister Olsson, Jiayi He, Therese Stark, Zuzanna Sadowska, Göran Molin, Siv Ahrné, Beatrix Alsanius, Åsa Håkansson

**Affiliations:** ^1^ Department of Food Technology, Engineering and Nutrition Lund University Lund Sweden; ^2^ Department of Biosystems and Technology Swedish University of Agricultural Sciences Alnarp Sweden

**Keywords:** *E. coli*, leafy green vegetables, ready‐to‐eat, rinsing, water bath

## Abstract

Customer demands for fresh salads are increasing, but leafy green vegetables have also been linked to food‐borne illness due to pathogens such as *Escherichia coli* O157:H7. As a safety measure, consumers often wash leafy vegetables in water before consumption. In this study, we analyzed the efficiency of household washing to reduce the bacterial content. Romaine lettuce and ready‐to‐eat mixed salad were washed several times in flowing water at different rates and by immersing the leaves in water. Lettuce was also inoculated with *E. coli* before washing. Only washing in a high flow rate (8 L/min) resulted in statistically significant reductions (*p* < .05), “Total aerobic count” was reduced by 80%, and *Enterobacteriaceae* count was reduced by 68% after the first rinse. The number of contaminating *E. coli* was not significantly reduced. The dominating part of the culturable microbiota of the washed lettuce was identified by rRNA 16S sequencing of randomly picked colonies. The majority belonged to *Pseudomonadaceae*, but isolates from *Enterobacteriaceae* and *Staphylococcaceaceae* were also frequently found. This study shows the inefficiency of tap water washing methods available for the consumer when it comes to removal of bacteria from lettuce. Even after washing, the lettuce contained high levels of bacteria that in a high dose and under certain circumstances may constitute a health risk.

## 1 INTRODUCTION

Lettuce is a healthy component in our diet, and ready‐to‐eat lettuce in plastic bags has become a widespread and convenient alternative to include “greens” on an everyday basis.

The microbiota of leafy green vegetables is varied and can contain pathogens or potential pathogens (Jackson, Randolph, Osborn, & Tyler, 2013), and at a number of occasions this produce has been involved in outbreaks of food‐borne illness (Doyle & Erickson, 2008; Painter et al., 2013). Leafy green vegetables are sensitive to bacterial contamination and multiplication. Bacteria can be transferred to the lettuce from soil, manure, water, equipment, and people (Castro‐Rosas et al., 2012; Doyle & Erickson, 2008). Processing can induce leaf damage that promotes bacterial growth (Gleeson & O'Beirne, 2005). Additionally, as these products are eaten raw, handling has to be done with meticulous care both in the production chain and at home.

Attempts to improve the microbial status of lettuce products have been performed through both physical and chemical means. The effectiveness of irradiation on human pathogens is limited, the consumer acceptance of irradiated foods remains low (Parish et al., 2003), and the authorities in many countries remain skeptical. Ultrasound can kill some bacteria by intracellular cavitation but the method is not very efficient, especially not in the presence of solids (Gil, Selma, López‐Gálvez, & Allende, 2009). Washing with the addition of sanitation agents reduce bacterial levels, but it also raises concern about negative, long‐term health effects and about environmental consequences. Chemicals like chlorine have been banned in some countries in Europe, for example, Sweden, Germany, and Switzerland (Gil et al., 2009; Parish et al., 2003). Unfortunately, most of the sanitizing solutions applied in industrial handling, even if efficient at first, fail to retain the effect when the product reaches the customer due to rapid bacterial regrowth (Allende, Selma, López‐Gálvez, Villaescusa, & Gil, 2008).

Thus, the question remains: Can the consumer influence the situation by household washing with clean water? This question may at first glance appear self‐evident, but in fact, when it comes to the bacterial reduction by household washing, very few data from the literature is available. We have only found one study where *Listeria* inoculated lettuce leaves were immersed in water which reduces the number of *Listeria* (Natsou, Rhoades, Smirniotis, Makri, & Kontominas, 2012). However, the effect of washing on the native flora was not considered. Otherwise, previous studies have focused on the efficiency of commercially available sanitation agents, and the risk of cross‐contamination of specific pathogens in industrial and domestic environments (Baur, Klaiber, Wei, Hammes, & Carle, 2005; Beuchat, 1998; Jensen, Friedrich, Harris, Danyluk, & Schaffner, 2015; Nou & Lou, 2010; Vijayakumar, 2002). The aim of the present study is to give an answer to the question if household washing of lettuce can be expected to have any effect on the bacterial load in general, and on contaminating *E .coli* in particular.

## 2 MATERIALS AND METHODS

### 2.1 Lettuce

Six bags of whole romaine lettuce heads (*Lactuca sativa*) and 18 bags of ready‐to‐eat mixed salad containing friseé (*Cichorium endivia* var. *crispum*), red salad (*Lactuca sativa*), and red mangold (*Beta vulgaris*) were purchased at a supermarket in Lund, Sweden in February 2016 and brought directly to the laboratory. Samples were of the same brand throughout all experiments. For each washing procedure, the samples were purchased on the same day, and originated from the same lot. The core and outer leaves from the romaine lettuce were removed and discarded before the remaining leaves were cut into square pieces (approx 4 × 4 cm) (as it often is done in the household) prior to analysis. Nothing was discarded from the ready‐to‐eat mixed salad.

### 2.2 Washing

Before washing, one sample of 10 g from each package was taken out for analysis. The remaining lettuce was then washed by two different procedures, rinsing in a colander under flowing water, and by immersion in a container with water. Both washing procedures were performed in sets of 6 packages. Rinsing was carried out on 3 sets; one set of ready‐to‐eat salad rinsed at a flow of 2 L/min, one set of ready‐to‐eat salad rinsed at a flow of 8 L/min and one set with romaine lettuce rinsed at a flow of 8 L/min. Immersion was carried out on 2 sets; one set of ready‐to‐eat salad and one set of romaine lettuce.

With rinsing, the contents were rinsed separately in colanders under a potable water tap; the salad mixtures were washed in running tap water for 20 s, and one sample of 10 g per package was thereafter taken out for analysis. The remaining leaves were rinsed 4 more times during 20 s each time, and after a total of 5 washes, another 10 g sample per package was taken out for analysis. This resulted in a total of 6 unwashed samples, 6 samples from the first wash (total wash water volume of 0.67 L) and 6 samples from the fifth wash (total wash water volume of 3.33 L) for each set.

With immersion, the content of 6 mixed salad packages of the same brand were washed in a container with 2 L potable water and shaken at 200 rpm for 30 s. One sample of 10 g per package was taken out for analysis and the remaining leaves were immersed 4 more times during 30 s each time, and after a total of 5 immersions, another 10 g sample per package was taken out for analysis. Between each immersion, the washing water was changed.

### 2.3 *Escherichia coli* inoculation of romaine lettuce

*E. coli* for inoculating romaine lettuce was prepared by transferring 1 μl of nonpathogenic *E. coli* CCUG 29300 (culture stored at −80°C in Hogness' freezing media (36 mmol/L K_2_HPO_4_, 13.2 mmol/L KH_2_PO_4_, 0.4 mmol/L MgSO_4_, 1.7 mmol/L Na_3_‐citrate, 6.8 mmol/L (NH_4_)_2_SO_4_, 4.4% (v/v) glycerol)) to 5 ml Tryptic Soy Broth (TSB) (Fluka, Missouri, USA) and incubating for 24 hr. After a subsequent centrifugation (4,600*g*, 5 min (Eppendorf Centrifuge 5804, Germany)), the pellet was washed twice with peptone water (0.85% NaCl and 0.1% bacteriological peptone (Oxoid, Basingstoke, UK)) and diluted to a concentration of log_10_ 6 CFU/ml (assessed by colony count and spectrophotometry (Novaspec II, Pharmacia, Sweden)).

Six separate heads of romaine lettuce (100 g per head) were cut according to previous description and to each 100 g sample, 1 ml inoculum and 200 ml potable tap water was added to give a final concentration of 5000 CFU/ml of *E. coli*. The bags were incubated at 4°C for 24 hr and then washed by immersion in water and sampled according to the procedure described above.

### 2.4 Microbial analysis

Viable count was performed on 10 g salad mixture or romaine lettuce samples. Each 10 g sample was homogenized in 90 ml peptone water for 2 min at high frequency on a Laboratory Blender Stomacher 400 (Seward Medical, London, UK). A diluted sample volume of 0.1 ml was spread with glass beads on duplicate plates. Brilliant *E. coli* Coliform selective Agar (ECBA) (Oxoid) for plate count of *E. coli*. Violet Red Bile Dextrose agar (VRBD) (Merck Millipore, Darmstadt, Germany) for count of *Enterobacteriaceae*, and Tryptic Soy Agar (TSA) (Fluka, Missouri, USA) was used for “total aerobic count”. The ECBA and VRBD plates were incubated at 37°C for 24 hr and the TSA plates were incubated at 30°C for 3 days.

In order to get an idea of the dominating culturable bacterial taxa of the total aerobic count and the *Enterobacteriaceae* count, 2 colonies per sample, or 36 colonies per set, in total 180 colonies, were randomly picked from countable plates of TSA and VRBD. Picked isolates were restreaked to purity, resuspended in freezing medium, and stored at −80°C until identification.

### 2.5 Sequencing of bacterial isolate DNA

Picked isolates were inoculated on TSA (Fluka) and incubated at 30°C for 3 days. Approximately log_10_ 8 cells could be harvested from each culture. The cells were suspended in 0.5 ml physiological saline followed by bead beating on an Eppendorf Mixer (model 5432, Eppendorf, Hamburg, Germany) for 30 minutes. After centrifugation at 600*g* for 30 s, the supernatant was used as template DNA in the subsequent Polymerase Chain Reaction (PCR). An approximately 1500 bp long fragment of the rRNA gene (16S) was amplified using the forward primer ENV1 (5′‐AGAGTTTGATIITGGCTCAG‐3′) and the reverse primer ENV2 (5′‐CGG ITA CCT TGT TAC GAC TT‐3′) (Eurofins Genomics, Ebersberg, Germany). The PCR was performed according to the manufacturer's instructions with TopTaq DNA Polymerase (Qiagen, Netherlands). The total volume of the PCR reaction was 25 μl, consisting of 0.2 μmol/L of ENV1 and ENV2, 2,5 μl 10× TopTaq PCR Buffer (Tris‐HCl, KCl, (NH4)2SO4, 15 mmol/L MgCl2, pH 8.7), 200 μmol/L of each deoxyribonucleotide triphosphate (dNTP), 0.625 U of TopTaq DNA Polymerase and 10–20 ng of template DNA. The PCR was performed by a Mastercycler gradient (Eppendorf) under the following conditions: 95°C for 3 min, followed by 30 cycles of 94°C for 1 min, 50°C for 45 s, 72°C for 2 min and at the end, an additional extension at 72°C for 10 min was performed. The results were confirmed by gel electrophoresis (1.5% agarose for molecular biology (Sigma‐Aldrich, Munich, Germany)) in Tris Acetate‐EDTA buffer (Sigma‐Aldrich). The gel was run at 120 V for 60 min and stained with GelRed (Biotium, USA) according to manufacturer's instructions. The PCR products were sent for sequencing at Eurofins Genomics (Ebersberg, Germany) on an ABI 3130xl Genetic analyzer (Applied biosystems, Foster City, CA, USA) using ENV1 as sequencing primer. The sequenced genes were trimmed to between 590 and 788 bp depending on sequence quality and compared to type strain sequences at the Ribosomal Database project (RDP) by the Seqmatch software (May, 2016) (6).

### 2.6 Statistical analysis

The colony count data was analyzed using SigmaPlot version 13.0 (SPSS Inc., Chicago, USA). The differences between all groups were evaluated by Kruskal–Wallis one‐way ANOVA on ranks, followed by all‐pairwise‐multiple‐comparison Student‐Newmns‐Keuls Method. The differences between two experimental groups were assessed by a Mann–Whitney rank sum test. Results were considered statistically significant when *p* ≤ .05. Values are presented as median with 25^th^ and 75^th^ percentiles.

## 3 RESULTS

### 3.1 Microbial counts after washing

Total aerobic count of ready‐to‐eat mixed salad rinsing decreased significantly (*p* ≤ .01) from a median value of 7.2–6.7 log_10 _CFU/g after the first wash (Table 1) at the higher water flow of 8 L/min. The *Enterobacteriaceae* count was reduced (*p* ≤ .05) from 5.7 to 5.2 log_10 _CFU/g after the first wash, but a greater level of significance (*p* ≤ .01) was achieved after the fifth wash, at 4.2 log_10 _CFU/g (Table 1).

**Table 1:** Viable counts of ready‐to‐eat mixed salad and romaine lettuce washed under the water tap and after immersion in a container with water

Washing method	Total aerobic count^a^			*Enterobacteriaceae* count^a^		
	Unwashed	1st wash	5th wash	Unwashed	1st wash	5th wash
Rinsing	Ready‐to‐eat mixed salad					
(2 L/min)	7.2 (6.4–7.9)	6.7 (6.3–7.0)	6.8 (5.6–7.3)	5.0 (4.6–5.5)	4.7 (4.0–5.2)	4.7 (4.0–5.0)
Rinsing	Ready‐to‐eat mixed salad					
(8 L/min)	8.3 (8.0–8.5)	7.6 (7.4–7.8)^c^	7.3 (7.1–7.5)^c^	5.7 (5.4–5.8)	5.2 (4.9–5.4)^b^	4.2 (3.9–4.5)^c^
	Romaine lettuce					
	5.0 (4.6–5.4)	4.1 (3.9–4.3)^b^	3.6 (3.1–4.0)^c^	3.4 (2.1–4.6)	2.7 (2.0–3.3)	2.2 (2.0–2.7)
Immersion	Ready‐to‐eat mixed salad					
	7.2 (6.5–7.6)	7.1 (5.8–7.3)	6.7 (5.5–7.0)	5.1 (4.8–5.7)	4.8 (4.5–5.2)	4.3 (3.2–5.2)
	Romaine lettuce					
	6.1 (5.4–6.6)	5.8 (5.1–6.3)	5.4 (4.8–5.1)	5.0 (4.5–5.4)	4.5 (4.1–4.8)	4.3 (3.5–4.4)

a Counts expressed as median log_10_ CFU/g salad of six replicates with intermedian range (25‐75%).

b Indicates *p* ≤ 0.05 compared to unwashed.

c indicates *p* ≤ 0.01 compared to unwashed in the same row.

The total aerobic count of romaine lettuce was also reduced significantly at 8 L/min (5.0 log_10 _CFU/g for unwashed lettuce, 4.1 log_10 _CFU/g after the first wash, and 3.6 log_10 _CFU/g after the fifth wash), but no significant reduction in *Enterobacteriaceae* count could be detected (Table 1).

In contrast, when washing at a lower water flow (2 L/min), neither the aerobic count nor the *Enterobacteriaceae* count was reduced significantly on ready‐to‐eat mixed salad. Similarly, after immersion of the produce in water, no statistical differences could be seen (Table 1).

Unwashed, *E. coli* inoculated romaine lettuce harbored a median value of 5.0 (4.4–5.4) log_10 _CFU *E. coli*/g, after the first wash 4.2 (4.1–5.1) log_10 _CFU/g and after the fifth wash 3.9 (3.5–4.6) log_10 _CFU/g (intermedian range 25–75% presented within parenthesis). This reduction was not statistically significant.

### 3.2 Identification

The identities of the rRNA gene (16S) sequenced isolates from mixed salad are shown in Table 2. Bacteria from the *Pseudomonaceae* and *Shewanellaceae* families were isolated from TSA both before and after rinsing. *Brevundimonas*,* Erwinia*,* Micrococcus*, and *Yersinia* species were found after rinsing. On VRBD, *Enterobacteriaceae* species such as *Pantoea* and *Serratia* were found both before and after rinsing.

**Table 2:** Putative identification by 16S rRNA gene sequencing of isolates from ready‐to‐eat mixed salad picked from the countable plate of the total aerobic plate (TSA) and *Enterobacteriaceae* plate (VRBD)

Closest type strain^a^	Similarity (%)	TSA^b^	VRBD^c^
Ready‐to‐eat mixed salad			
*Bacillus anthracis* (T) AE016877	100.0	1^e^	—
*Bacillus cereus* (T) AB190217	100.0	1^d^	1^e^
*Bacillus methylotrophicus* (T) EU194897	96.8	—	1^e^
*Brevundimonas vesicularis* (T) AJ227780	100.0	1^e^	—
*Curtobacterium herbarum* (T) AJ310413	99.7	1^d^	—
*Erwinia persicina* (T) U80205	100.0	1^e^	—
*Micrococcus luteus* (T) AJ536198	98.4	1^e^	—
*Pantoea agglomerans* (T) AJ233423	98.0	1^d^	1^d^
*Pantoea eucalypti* (T) EF688009	98.2	—	3^d^+3^e^
*Pantoea vagans* (T) EF688012	100.0	—	1^d^+2^e^
*Pseudomonas azotoformans* (T) D84009	99.8	1^e^	—
*Pseudomonas constantinii* (T) HAMBI 2444	100.0	1^d^	—
*Pseudomonas gessardii* (T) AF074384	99.8	1^e^	—
*Pseudomonas grimontii* (T) AF268029	100.0	1^e^	—
*Pseudomonas koreensis* (T) AF468452	100.0	1^d^	—
*Pseudomonas libanensis* (T) AF057645	100.0	1^d^	—
*Pseudomonas marginalis* (T) Z76663	100.0	1^e^	—
*Pseudomonas psychrophila* (T) AB041885	99.7	1^d^+1^e^	—
*Rahnella aquatilis* (T) AJ233426	99.9	2^d^	—
*Serratia ficaria* (T) AJ233428	94.3	—	1^d^
*Serratia liquefaciens* (T) AJ306725	99.7	—	1^e^
*Shewanella putrefaciens* (T) X81623	100.0	1^d^+1^e^	—
*Staphylococcus warneri* (T) L37603	97.8	—	1^e^
*Yersinia aldovae* (T) AF366376	99.6	1^e^	—
Romaine lettuce			
*Arthrobacter polychromogenes* (T) X80741	99.5	2^d^	—
*Bacillus anthracis* (T) AB190217	99.4	1^d^	1^e^
*Bacillus cereus* (T) AE016877	100.0	1^e^	—
*Bacillus safensis* (T) AF34854	100.0	1^e^	—
*Microbacterium phyllosphaerae* (T) AJ277840	99.8	1^e^	—
*Pantoea agglomerans* (T) AJ233423	98.1	2^d^	2^e^
*Pantoea eucalypti* (T) EF688009	96.0	1^d^	1^d^
*Pantoea vagans* (T) EF688012	97.0	1^e^	—
*Pseudomonas frederiksbergensis* (T) AJ249382	99.9	—	1^d^
*Pseudomonas koreensis* (T) AF468452	99.8	1^d^	—
*Staphylococcus warneri* (T) L37603	100.0	—	1^e^
*Stenotrophomonas chelatiphaga* (T) EU573216	99.7	1^d^	—

a Closest type strain (T) according to RDP data base, Seqmatch software(5).

b Number of isolates from TSA.

c Number of isolates from VRBD.

d isolate found before washing (unwashed).

e isolate found after washing (washed).

In the immersion experiment on mixed salad, bacteria from the *Bacillaceae* and *Pseudomonadaceae* families were found on TSA both before and after water bath washing. *Rahnella* and *Curtobacterium* were found on TSA only before washing. *Enterobacteriaceae* isolates were found on VRBD both before and after the water bath washing.

In the rinsing experiment of romaine lettuce, bacteria from the *Bacillaceae* and *Enterobacteriaceae* were found on TSA both before and after rinsing (Table 2). Members of the families *Micrococcacea*,* Xanthomonadaceae* and *Pseudomonadaceae* were found before rinsing, and *Microbacteriaceae* and *Staphylococcaceae* were found after rinsing.

## 4 DISCUSSION

In this study, two washing procedures feasible to perform under household conditions have been evaluated by their efficiency to remove bacteria from ready‐to‐eat mixed salad and from unwashed romaine lettuce.

Washing of mixed salad under a water tap at a flow rate of 8 L/min significantly reduced both total aerobic count and *Enterobacteriaceae* count, and this washing method also reduced the total aerobic count of romaine lettuce. Notably, the repeated washing steps under the water tap gave further significant reductions of both total aerobic count and *Enterobacteriaceae* count on both mixed salad and romaine lettuce. The non‐significant reductions of *Enterobacteriaceae* on romaine lettuce were probably due to the high variation in starting values between the samples, and an overall low concentration of *Enterobacteriaceae*. The washing flow of 8 L/min is probably in the higher range for household washing. At this water flow, the structural appearance of the leaves in the mixed salad were somewhat negatively affected, however, the romaine lettuce appeared tougher and more resistant to the force of running water.

At the more gentle water flow of 2 L/min, there was no significant decrease in the bacterial load, and the same was true with the method of washing in water bath. Only the higher washing flow of 8 L/min yielded significant reductions in bacterial content, indicating that the detachment of microorganisms is dependent on the mechanical force that is applied. The attachment of microorganisms seems to be of a simple physical entrapment nature, and independent on whether the cells are alive or dead (Solomon & Matthews, 2005).

All samples were washed five times, with a duration of 20 s each time. All five washes were needed for the total aerobic count to decrease one log_10_ unit of the ready‐to‐eat mixed salad, while on the romaine lettuce only one wash was needed to reach the same result. This might be due to the higher initial counts of the ready‐to‐eat salad, thus a log_10_ reduction is harder to achieve. Alternatively, bacteria colonizing the ready‐to‐eat salad were attached more firmly to the leaves than the bacteria on romaine salad. The bacterial community may vary due to morphological and chemical differences between lettuce genera and it is known that some microorganisms attach preferentially to cut edges, and are able to internalize the leaf tissue (Allende et al., 2008; Hunter, Hand, Pink, Whipps, & Bending, 2010; Jackson et al., 2013; Takeuchi & Frank, 2000). Pathogens that have internalized are known to be more resistant against sanitation agents and washing by physical means (Takeuchi & Frank, 2000). It is also worth noting that the initial values (before washing) of total count on romaine lettuce were 3 log_10_ units lower than for the mixed salad (Table 1). The reason to that might be the damage caused to the mixed salad during processing, which increases the physical deterioration and enhances microbial growth (Allende et al., 2008; Rico, Martín‐Diana, Barat, & Barry‐Ryan, 2007).

Even though the bacterial content of both lettuce products was reduced by approximately one log_10_ unit by the highest water flow, the reduction still left the lettuce with high amounts of bacteria. This is probably due to the high initial values, especially from the mixed salad with an *Enterobacteriaceae* unwashed count of approximately log_10_ 5 CFU/g. However, the *Enterobacteriaceae* counts can be considered a bit too high due to what the sequencing results shows. Of the isolates from VRBD on mixed salad and romaine lettuce, many were not identified as *Enterobacteriaceae* (Table 2).

From the sequencing results (Table 2) it can also be seen that it is gram negative bacteria that dominate, mostly α‐, γ‐proteobacteria together with a few gram positive bacteria such as *Bacillus* and *Actinobacteria*, supporting current research (Hunter et al., 2010; Jackson et al., 2013; Lopez‐Velasco, Carder, Welbaum, & Ponder, 2013). *Shewanella putrefaciens* is not commonly found on green leaves, but associated with spoilage of fish (Gram & Huss, 1996). It was therefore somewhat surprising to find it in the mixed salad (Table 2). However, *Shewanella* has been identified from hydroponic lettuce cultivation systems (Rivera, Vélez, Zayas, & Llamas, 2015) and its presence may indicate a marine source of irrigation water or contaminated processing surfaces (Bagge, Hjelm, Johansen, Huber, & Gram, 2001). *Shewanella* is known to cause problems by adhering strongly to surfaces through biofilm formation (Bagge et al., 2001). In this study it was found both before and after washing. A single isolate was putatively identified as *Bacillus anthracis*, however, the genetic variations of *Bacillus cereus*,* Bacillus anthracis*, and *Bacillus thuringensis* exist mainly on episomes rather than on the chromosome genes (Ash & Collins, 1992; Rein Carlson, Caugant, & Kolsto, 1994). Therefore, it is impossible to distinguish between these species purely with 16S rRNA gene sequencing.

Within the International Commission on Microbiological Specifications for Foods and the European Commission, the safety guidelines that exist for fresh‐cut lettuce are only specified for *E. coli* and *Salmonella* (European Commission, 2005; Health Protection Agency, 2009). None of these taxa were found in the present study, but the number of sequenced isolates was limited, and the viable counts point at high concentrations of bacteria. The sequencing results reveal that a substantial part of the flora consists of *Enterobacteriaceae*; a family including many members with pathogenic potential. A high dose of these types of bacteria might pose a risk for the consumer, especially to children, elderly and immunocompromised individuals. Besides, there are those of the members that are notorious carries of antibiotic resistance.

To simulate a possible *E. coli* contamination, either during production or processing, romaine lettuce was subjected to *E. coli* inoculation. The lettuce was incubated in a water bath with added *E. coli* at 5000 CFU/ml. After 24 hr at 4°C, the samples were immersed in potable water five times. The results showed that it was not possible to significantly lower the *E. coli* count by washing.

## 5 CONCLUSIONS

This study shows that even though the salad mix is already washed and ready to eat, it contains high amounts of viable bacteria belonging to the *Enterobacteriaceae* family, a family which includes several pathogenic taxa. Simulated household washing could at best reduce the total aerobic count and the *Enterobactreriaceae* count with 90% and 97%, respectively, which due to high loads still left the leafy vegetables with high amounts of bacteria.

The results of this study show the inefficiency of tap water washing methods available in the home without chemical additions to remove bacteria from lettuce below safe limits. This situation stresses the responsibility of producers and distributors to ensure the hygienic quality of the green produce.

## CONFLICT OF INTEREST

None declared.
